# Increased Langerhans cell accumulation after mycobacterial stimuli

**DOI:** 10.1111/j.1365-2559.2007.02848.x

**Published:** 2007-11

**Authors:** A Miranda, T P Amadeu, G Schueler, F B F Alvarenga, N Duppré, H Ferreira, J A C Nery, E N Sarno

**Affiliations:** 1 Leprosy Laboratory, Oswaldo Cruz Institute, Oswaldo Cruz Foundation Rio de Janeiro; 2 Department of Histology and Embryology, Biology Department, State University of Rio de Janeiro RJ, Brazil; 3 Department of Pathology and Laboratories, State University of Rio de Janeiro RJ, Brazil

**Keywords:** dendritic cells, infection, Langerhans cells, leprosy, *Mycobacterium leprae*

## Abstract

**Aims:**

To evaluate the role of Langerhans cells (LCs) in the local activation of leprosy lesions. LCs, acting as tolerance inducers and immune stimuli, are dendritic cells recently implicated in cutaneous homeostasis. The role of LCs in the defence against mycobacterial infection remains poorly understood.

**Methods and results:**

The number and distribution of CD1a+ skin cells and HLA-DR and intercellular adhesion molecule (ICAM)-1 expression were analysed in leprosy skin lesions and in delayed-type hypersensitivity (DTH) tests. The results showed a high number of LCs in tuberculin and lepromin tests, in tuberculoid lesions and in the epidermis and dermis during type I and II reactions. In multibacillary lesions, however, the number of LCs was consistently low in comparison with other groups. Increased numbers of LCs were accompanied by marked HLA-DR and ICAM-1 expression, suggesting a strong relationship between these immunological events.

**Conclusions:**

CD1a+ cells are implicated in the local immunological events taking place after mycobacterial stimuli and may account for the local activation of all types of reactional episodes in leprosy.

## Introduction

Leprosy is a chronic infectious disease whose clinical spectrum is dependent upon the immunological status of the patient. At the tuberculoid pole, patients demonstrate well-mounted cell-mediated immunity (CMI), which, although limiting the number of bacilli and lesions, accounts for the prominent impairment of the peripheral nerves. Conversely, the lepromatous pole is characterized by widespread skin lesions, unrestricted multiplication of bacilli and an impaired cellular immune response. Acute inflammatory episodes, designated ‘reactional episodes’ or ‘reactions’, complicate the clinical course of the disease and are responsible for even worse neurological damage to the peripheral nervous system.

Reactional episodes are acute inflammatory processes that interrupt the chronic, insidious course of leprosy. The mechanisms involved in triggering these processes remain unknown. Reactions are believed to represent the re-emergence or enhancement of CMI, which has been either lost or down-regulated during chronic infection. The mechanisms involved in this immune reactivation are of great interest and present an on-going challenge to immunologists.^1^

A previous study^2^ has shown a direct correlation between the number of T cells, CD1 and effective immunity. In this context, a greater number of CD1+ cells was found in the dermal infiltrate in tuberculoid and reversal reaction (RR) lesions, but not in erythema nodosum leprosum (ENL) lesions. Moreover, in the same study, the number of CD1a cells within the epidermis of lepromatous leprosy (LL) and tuberculoid lesions was not significantly different when compared with that found in normal skin.

CD1 belongs to a family of non-polymorphic, β_2_-microglobulin-associated, transmembrane glycoproteins that are structurally related to classical major histocompatibility complex antigen-presenting molecules.^3^–^5^ Recent studies have established the important role played by CD1 molecules in lipid antigen recognition by T cells. Mycobacterial cell wall components such as mycolic acid, lipoarabinomannan and others are presented by CD1a, B dendritic cells and are recognized by α, β T cells.^6^ It is known that Langerin exclusively expressed by LCs is an important antigen uptake receptor for the generation of such T cells.^7^

Comprising 1–3% of all epidermal cells, LCs are bone marrow-derived dendritic cells (DCs) asymmetrically distributed within the epidermis and other keratinized and non-keratinized squamous epithelia.^8^ LCs initiate immune responses by capturing and processing foreign antigens and then emigrating to the regional lymph nodes, where they present the processed antigen to naive T cells. It is believed that these cells return to the skin where they act as inflammatory cells.^8^,^9^

Langerhans cells have been implicated in the maintenance of cutaneous homeostasis by inducing tolerance as well as stimulating immune responses. In many skin diseases with an enhanced influx of T lymphocytes, the total number of LCs increases in the epidermis and decreases after exposure to ultraviolet rays or toxic substances.^10^,^11^

It has also been demonstrated that tumour necrosis factor (TNF), in addition to granulocyte macrophage–colony-stimulating factor and interleukin (IL)-1, are involved in the LC maturation process. TNF, for the most part produced in the skin in a number of inflammatory conditions, significantly increases the number of viable LCs while decreasing their apoptosis.^12^ In this same study, TNF-α induced LCs to produce interferon (IFN)-inducible protein 10/CXCL10, a Th1-attracting chemokine and IL-12p40.^12^ It has been consistently demonstrated that TNF is up-regulated in type I and type II reactions.^1^ However, the antigen and the immune T-cell subsets involved in the reactivation of CMI during these reactions are still unknown.

To obtain more knowledge of the role of epidermal LCs in the immune response to *Mycobacterium leprae*, the causative agent of leprosy, and the pathogenic mechanisms underlying *M. leprae*-induced immune responses in both types of leprosy reactions, a quantitative analysis of epidermal and dermal CD1a expression in the biopsy specimens of cutaneous leprosy lesions was performed using immunohistochemistry. These findings were compared with those observed in samples taken from two mycobacterial skin tests [purified protein derivative (PPD) and lepromin] widely known to represent local CMI against mycobacterial antigens.

## Patients and methods

### Patients

The study was carried out on skin biopsy specimens taken from the lesions of 94 leprosy patients from the Leprosy Laboratory, Department of Mycobacterioses, Oswaldo Cruz Institute, Rio de Janeiro, RJ, Brazil. Skin biopsy specimens (6 mm in diameter) containing both epidermis and dermis were obtained by the standard punch technique, following informed consent.

Patients were classified clinically according to the Ridley and Jopling criteria^13^ and those selected divided into four groups: (i) those with type I reaction (RR); (ii) those with type II reaction [ENL or erythema multiforme (EM)]; (iii) those with borderline tuberculoid leprosy (BT) and (iv) those with multibacillary leprosy [borderline leprosy (BL)/LL forms]. In addition, the biopsy specimens taken from lepromin and PPD+ tests (DTH group) were also included.

### Immunohistochemical analysis of CD1, HLA-DR and intercellular adhesion molecule-1 expression in leprosy skin lesions

Biopsy specimens were snap-frozen in liquid nitrogen until use. Serial sections (6 μm) were acetone fixed at 4°C for 10 min and blocked with normal horse serum for 30 min (Vector Laboratories, Burlingame, CA, USA) before incubation with monoclonal antibodies (mAbs). For assessment of LCs or keratinocytes expressing HLA-DR and intercellular adhesion molecule (ICAM)-1, sections were, respectively, incubated with mouse mAbs antihuman CD1 (1:50), antihuman HLA-DR (1:25 dilution; Becton Dickinson, Mountain View, CA, USA) or antihuman ICAM-1 (CD54, 1:25; Immunotech, Marseilles, France) overnight at 4°C. For revelation, biotinylated horse antimouse IgG (1:150; 60 min) and Vectastain ABC Kit (Vector Laboratories) were used. As a chromogen, 3-amino-9-ethylcarbazole was used and slides were counterstained with haematoxylin. Negative controls were performed after omitting the primary antibody; no labelling was observed.

### Quantification of CD1 and HLA-DR expression in leprosy skin lesions

The number of epidermal and dermal cells expressing CD1 was evaluated using a conventional optical microscope. In the epidermis, the total number of CD1+ cells was counted using a large field (×40) objective and the average CD1+ cell count per field was calculated. In the dermis, the number of CD1+ cells was evaluated by counting their total number in the whole biopsy section. In each group, data are expressed as median ± 95% confidence interval (CI).

The relative density of keratinocytes expressing HLA-DR was semiquantitatively determined, as follows: (i) absence of stained keratinocytes; (ii) when 25% of keratinocytes were HLA-DR+; (iii) when 50–75% of keratinocytes were HLA-DR+ or (iv) when >75% of keratinocytes were HLA-DR+. In each group, data are expressed as median ± 95% CI.

In addition, biopsy specimens of all patients with type II reaction, BT group, multibacillary leprosy (BL/LL) group and DTH group were labelled with anti-ICAM-1. The expression by keratinocytes was qualitatively evaluated as positive or negative.

### Statistical analysis

A non-parametric Mann–Whitney test was used to analyse epidermal and dermal CD1+ cells. Fisher’s test was used to analyse the semiquantitative density of keratinocytes expressing HLA-DR. Graph Pad Instat version 3.01 software (GraphPad Software Inc., San Diego, CA, USA) was used and *P* < 0.05 was considered to be statistically significant.

## Results

### CD1 expression in cutaneous leprosy lesions

Immunohistochemistry for CD1a molecules was performed on biopsy specimens of skin lesions from 12 patients with type I reaction, 33 with type II reaction, 10 BT patients, 19 multibacillary patients and 20 lepromin-positive (*n* = 6) or PPD+ (*n* = 14) tests.

**Figure 1:**
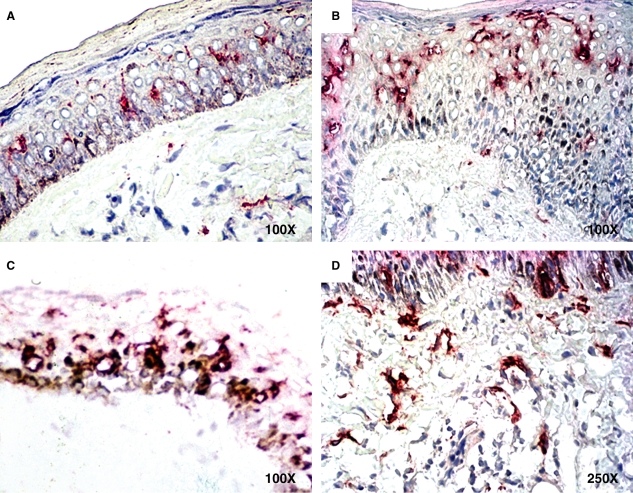
CD1 expression in leprosy skin lesions. **A**, a few positive cells in suprabasal localization of flattened epidermis of lepromatous leprosy/borderline leprosy patients. **B**, dendritic cells in upper layers of the thickened epidermis in a type II reaction patient. **C**, marked positive dendritic cells in a type I reaction patient. **D**, numerous positive cells in the dermis of a type II reaction patient. The original magnification is shown.

All epidermal CD1a+ cells presented a similar neuronal-like aspect observed in dermal CD1a+ cells and were most often localized in the suprabasal layers between keratinocytes (Figure 1A–C). No difference was observed in the BL/LL group in comparison with normal skin.

**Figure 2:**
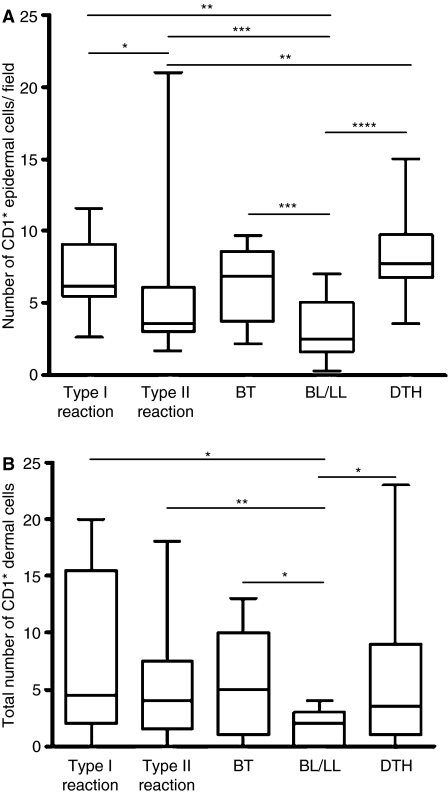
Quantification of CD1 expression in leprosy lesions in all groups studied [biopsy specimens of type I and type II reactions; tuberculoid (BT) and lepromatous (BL); and lepromin and purified protein derivative-positive tests (DTH)]. **A**, number of CD1+ epidermal cells per field. **B**, total number of CD+ dermal cells. Median ± 95% CI. **A**, *P < 0.05; **P < 0.0005; ***P < 0.005; ****P < 0.0001. **B**, *P < 0.05; **P < 0.005.

Quantification of epidermal CD1a+ cells showed a 24% increase in patients with a type I reaction (6.2 ± 1.5) in comparison with patients with a type II reaction (3.6 ± 1.5; *P* < 0.05; Figure 2A). When patients with type I and type II reactions were compared with the BT group (6.9 ± 1.6), no differences were observed. However, when compared with the BL/LL group (2.5 ± 0.9), the number of epidermal CD1a+ cells was 135% and 90% higher (*P* < 0.0005 and *P* < 0.005), respectively (Figure 2A). Data showed that the number of epidermal CD1a+ cells was 121% higher in the BT group (6.9 ± 1.6) than in the BL/LL group (2.5 ± 0.9; *P* < 0.005; Figure 2A). Moreover, numbers of epidermal CD1a+ cells were 180% and 51% higher, respectively, in the DTH positive group (7.8 ± 1.2) in comparison with the BL/LL group (2.5 ± 0.9; *P* < 0.0001) and with type II reactional patients (3.6 ± 1.5; *P* < 0.0005; Figure 2A).

Dermal cells expressing CD1a were also evaluated and quantified. These cells were aggregated in the papillary dermis which is often localized around superficial capillaries (Figure 1D). Although the dermal CD1a+ cells also varied in number, their distribution followed the same pattern as that observed in the epidermis. That is to say that, on the one hand, the cell count was higher when tissue DTH features were prominent, as seen in the BT and DTH groups and in type I reaction, but moderate in type II reaction and close to zero in the BL/LL group (Figure 1).

When patients with type I and type II reactions (4.5 ± 4.3 and 4.0 ± 1.7, respectively) were compared with the BL/LL group (2.0 ± 0.7), the total number of dermal CD1a+ cells was 387% and 225% higher (*P* < 0.05 and *P* < 0.005), respectively (Figure 2B). The number of dermal CD1a+ cells per biopsy specimen was 250% higher in the BT group (5.0 ± 2.8) than in the BL/LL group (2.0 ± 0.7; *P* < 0.05; Figure 2B). In addition, the total number of dermal CD1a+ cells was 263% higher in the DTH+ group (3.5 ± 2.9) when compared with the BL/LL group (2.0 ± 0.7; *P* < 0.05; Figure 2B).

### HLA-DR expression in cutaneous leprosy lesions

To confirm the simultaneous immune activation of the epidermis in leprosy, lesions expressing HLA-DR and ICAM-1 molecules were analysed in the keratinocytes in all samples. Immunohistochemical analysis of HLA-DR expression was performed on biopsy specimens of skin lesions from 12 patients with type I reaction, 33 with type II reaction, 10 from the BT group and 19 from the BL/LL group.

**Figure 3:**
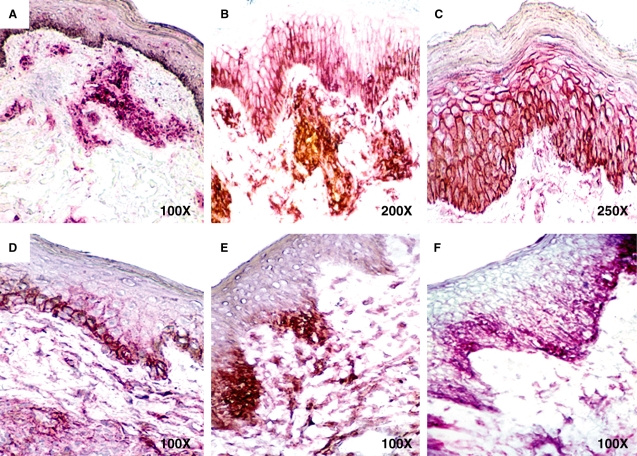
HLA-DR and intercellular adhesion molecule (ICAM)-1 expression in leprosy skin lesions. Figures are representative of HLA-DR (**A**–**C**) and ICAM-1 (**D**–**F**). **A**, only the dermal infiltrates are labelled in a type II reaction biopsy. **B**, fifty per cent of keratinocytes are labelled in a borderline tuberculoid patient. **C**, marked expression in type I reaction. **D**, labelled cells along the basal layer (type II reaction). **E**, two marked foci in type II reaction. **F**, the basal layer and addictional foci are stained in a delayed-type hypersensitivity group biopsy specimen. The original magnification is shown.

As previously stated regarding the presence of LCs, positivity to HLD-DR within the epidermis was also marked in all cases in which CMI to *M. leprae* was clearly developed, but was mild or absent when effective CMI was lacking, as in the BL/LL group. In normal skin, only constitutively positive LCs expressed HLA-DR (Figure 3A–C).

**Figure 4:**
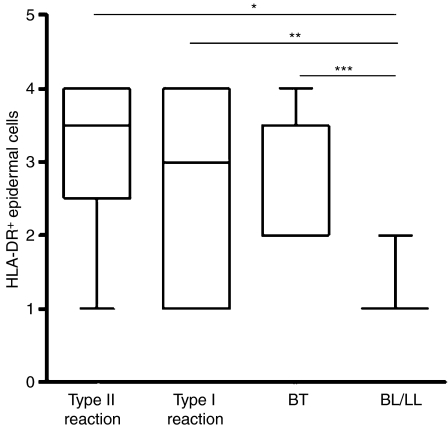
Relative density of keratinocytes expressing HLA-DR in leprosy lesions in all groups studied [biopsy specimens of type I and type II reactions; tuberculoid (BT) and lepromatous (BL)]. Semiquantitative score: absence of stained keratinocyte: (1) 25% of keratinocytes are HLA-DR+; (2) 50–75% of keratinocytes are HLA-DR+; (3) >75% of keratinocytes are HLA-DR+; (4) median ± 95% CI. *P < 0.001; **P < 0.0001; ***P < 0.05.

Semiquantitative analysis of HLA-DR+ epidermal cells showed that the number of keratinocytes expressing HLA-DR was not different for patients with either type I (3.0 ± 0.7) or type II reactions (3.5 ± 0.4). When these patients were compared with the BT group (2.0 ± 0.8), the density of HLA-DR+ keratinocytes was less in the BL/LL group (1.0 ± 0.2) than in patients with type I (3.0 ± 0.7; *P* < 0.001) or type II reactions (3.5 ± 0.4; *P* < 0.0001), or in the BT group (2.0 ± 0.8; *P* < 0.05; Figure 4).

### ICAM-1 expression in cutaneous leprosy lesions

An evaluation was also made of the expression of ICAM-1 on the surface of keratinocytes in the skin lesions of 33 patients with type II reaction, 10 BT patients, 19 BL/LL patients and 20 DTH patients. Normal skin biopsy specimens were used as controls. In normal skin and BL/LL cutaneous lesions, ICAM-1 was not expressed. The BT and the DTH groups showed mild to moderate expression of ICAM-1, which rarely included the entire thickness of the epidermis, but was, conversely, restricted to two or three foci. In type II reaction, mild ICAM-1 expression, confined to basal layers or to no more than two foci of keratinocytes, could be detected (Figure 3D–F) in each specimen.

## Discussion

The skin provides the initial barrier of protection against the invasion of pathogens and harmful stimulants into the body. In this study, LCs, as defined by their positivity for CD1a, were found in varying concentrations within the epidermis, papillary dermis and granulomas in all samples tested. In the absence of any other known antigen stimulation, it can be assumed that the higher concentration of LCs in reactional episodes is the result of the same immunological events underway in tuberculoid lesions and mycobacterial positive skin tests. As previously reported by Sieling *et al.,*^2^ the number of LCs in the epidermis within leprosy lesions is closely related to the extent of the cutaneous cell-mediated immune response. This increase would be the result of the recruitment of CD1 precursors from bone marrow or the adjacent skin.^14^

In our study, a significant increase in the number of LCs was observed in both RR and ENL reactional lesions, in which the LC mean in the epidermis was similar to that found in tuberculoid lesions. Interestingly, the number of LCs detected in reactional lesions was also comparable to that detected in the DTH lesions (lepromin and PPD), suggesting that this effect is related to mycobacterial antigens and is not leprosy specific. It could be hypothesized that even in multibacillary patients in the throes of reaction, the new *M. leprae* antigens reaching the skin activate the cutaneous DCs, which, in turn, trigger new migratory waves of inflammatory immune cells responsible for the appearance of new lesions. In addition to the LC increase, reactional lesions are characterized by a dense infiltration of newly recruited, activated macrophages, lymphocytes and polymorphonuclear leucocytes, which progressively disappear after corticosteroid and thalidomide (ENL) therapy. Previous studies have suggested that RR is triggered by LC activation.^15^ The current study has extended this hypothesis to ENL lesions. Moreover, it has recently been demonstrated that LC-like DCs are able to present non-peptide antigens of *M. leprae* in a CD1a-restricted manner. A CD1a-restricted T cell that recognizes mycobacterial antigens has been identified as a CD8+ T cell.^7^,^16^ The role of this T subset in reactional activation in leprosy requires clarification. Some intriguing data from the present study concern the high number of LCs detected in the epidermis of type II reactional patients whether the lesions were ENL or EM. Although some studies have demonstrated a few aspects of CMI reactivation during type II reaction, our study has strengthened this hypothesis. The histopathological findings of type II reaction in fact differ from those seen in RR, whereas vascular changes and polymorphonuclear infiltration predominate in type II reaction. As in many other inflammatory skin diseases, LCs trigger the immune response, but the pathological aspects of the lesions depend on the cytokine profile secreted by the inflammatory cells attracted to the lesion site.

It is now assumed that, during a type II reaction, higher levels of TNF are secreted locally. TNF-α has been implicated in the mobilization of LC precursors from the bone marrow, as well as the maturation of DCs. *In vitro* studies have shown that TNF-α significantly increases the number of viable LCs by decreasing their spontaneous apoptosis.^17^ On the other hand, TNF and IL-1B have been reported to increase LC migration, in this way reducing the number of cells in the epidermis and increasing cell numbers in the dermis.^18^ It is also known that LCs can produce large amounts of IL-1 and TNF in response to exogenous stimuli and cytokines.^19^ High levels of mRNA TNF expression have been demonstrated in both types of reactional lesions.^12^,^20^

The mechanisms regulating the expression of HLA-DR and ICAM-1 in reactional lesions are not well understood. In normal skin, KCs express neither HLA-DR nor ICAM-1 and LCs are the only HLA-DR+ cells. However, these molecules have been repeatedly shown in the epidermis covering tuberculoid and reactional lesions. The increased expression of these molecules in both types of reaction has confirmed previous studies demonstrating increased IFN-γ mRNA and TNF mRNA expression in reactional lesions.^21^ The correlation between epidermal and dermal LC numbers suggests that a dynamic flux of LC streams from the epidermis toward the dermal venules coexists both in reactional lesions and in DTH skin tests. Variations in LC numbers in the skin have also been found in cutaneous leishmaniasis. Higher LC numbers have been detected in localized cutaneous lesions in contrast to the defensive forms of the disease.^22^

In the present study, higher LC numbers in both the epidermis and dermis were detected in conditions known to have an established CMI, such as in PPD+ and lepromin-positive tests. In both tests, the injection of mycobacterial antigens induced a local immune response in previously sensitized individuals.

The tuberculin reaction has been considered the prototype of a DTH and has been useful in determining the cell components of CMI *in situ*. Injection of PPD into the skin of lepromatous leprosy patients leads to an emigration of IFN-γ secretion cells into the dermis, induction of HLA class II by KC, and accumulation of LCs in the dermis, accompanied by a reduced bacillary load.^23^ Lepromin reaction has been used as an important tool to differentiate between tuberculoid and lepromatous patients, since only the former are lepromin positive.

In this study, LC participation in response to mycobacterial antigens was confirmed by increasing LC numbers whenever effective CMI was observed in tissue biopsy specimens. The similar results observed in RR and ENL strongly support shared common mechanisms of LC activation. The difference between lepromatous patients and those groups with conspicuous CMI was statistically significant. The increase in LC numbers was accompanied by HLA-DR and ICAM-1 expression, suggesting a close relationship between these immunological events. The immunohistochemical approach using the antibody to CD1a may therefore be a useful tool in evaluating the immunological status of leprosy patients because of its ability to shed more light on the pathogenic mechanisms involved in RR and ENL.

## Abbreviations:

BL: borderline leprosy
BT: borderline tuberculoid
CI: confidence interval
CMI: cell-mediated immunity
DC: dendritic cell
DTH: delayed-type hypersensitivity
EM: erythema multiforme
ENL: erythema nodosum leprosum
ICAM: intercellular adhesion molecule
IFN: interferon
IL: interleukin
LL: lepromatous leprosy
mAb: monoclonal antibody
PPD: purified protein derivative
RR: reversal reaction
TNF: tumour necrosis factor
